# In-Use Stability and Functional Efficacy of a Monoclonal Antibody Cocktail (TwinRab™) for Rabies Post-exposure Prophylaxis

**DOI:** 10.7759/cureus.98418

**Published:** 2025-12-03

**Authors:** Pankaj Kalita, Sanjeev Kumar, Sanjay Bandyopadhyay, Chintan Patel, Mihir Patel, Mr. Swagat Soni, Hiral Patel, Tushar Patel, Niraj Sakhrani, Trayambak Dutta, Manish Mahajan

**Affiliations:** 1 Biotechnology, Zydus Lifesciences, Ahmedabad, IND; 2 Infectious Disease, Zydus Lifesciences, Ahmedabad, IND; 3 Medical Affairs, Zydus Lifesciences, Ahmedabad, IND

**Keywords:** monoclonal antibodies, post-exposure prophylaxis (pep), rabies, rabies immunoglobulin, twinrab™

## Abstract

Rabies remains a major public health problem in India, accounting for nearly one-third of global human deaths each year. Limited access to human and equine rabies immunoglobulins (RIGs) continues to restrict effective post-exposure prophylaxis (PEP) coverage, particularly in resource-limited settings. TwinRab™, a two-antibody monoclonal cocktail containing docaravimab and miromavimab, was developed as a standardized, scalable alternative to plasma-derived RIGs. This study evaluated the in-use stability and biological efficacy of TwinRab™ under simulated field-use conditions. In the in vitro arm, TwinRab™ samples from 300 IU/mL and 600 IU/mL batches were diluted in 0.9% sodium chloride to obtain twofold, fivefold, and 10-fold dilutions and stored at room temperature (25 ± 2 °C) for 4-24 hours. Potency was measured using the rapid fluorescent focus inhibition test (RFFIT) against the CVS-11 rabies virus strain. In the in vivo arm, undiluted TwinRab™ was administered six or 24 hours after viral challenge in a Syrian hamster model, and survival outcomes were compared with human RIG (Kamrab). TwinRab™ retained its neutralizing potency after dilution and ambient storage for up to 24 hours. The 600 IU/mL batch showed no significant loss of activity across all dilutions (p > 0.05), while minor but statistically significant differences were noted at higher dilutions of the 300 IU/mL batch (p < 0.05). In vivo, TwinRab™ achieved 90% survival when administered six hours post-challenge and 70% when administered after 24 hours, demonstrating comparable efficacy to Kamrab. These findings confirm that TwinRab™ maintains stability and functional efficacy under conditions that simulate routine clinical practice. Its thermostability, potency, and protective efficacy highlight its potential as a field-adaptable, next-generation alternative to conventional RIGs for PEP, particularly in high-burden regions with limited cold-chain infrastructure.

## Introduction

Rabies is a viral zoonotic disease that causes acute, progressive encephalitis and is almost invariably fatal once clinical symptoms appear. Globally, rabies accounts for an estimated 59,000 human deaths annually, with nearly 35% of these occurring in India [1,2]. The majority of fatalities result from dog bites in rural or underserved areas, where access to timely and complete post-exposure prophylaxis (PEP) remains limited. Despite the availability of effective vaccines and immunoglobulins, India continues to face challenges in achieving adequate PEP coverage due to infrastructure gaps, high costs, and supply chain limitations [3-5].

The World Health Organization (WHO) recommends combined active immunization with rabies vaccine and passive immunization using rabies immunoglobulin (RIG) for category III exposures [6]. However, access to human RIG (HRIG) and equine RIG (ERIG) in India remains restricted, particularly in the public health sector [7]. HRIG is expensive and dependent on human plasma donors, while ERIG - though more widely available - shows variable potency and carries a higher risk of hypersensitivity reactions. Consequently, fewer than 2% of category III exposures in India receive the recommended RIG component of PEP [8].

To address these limitations, several anti-rabies monoclonal antibodies (mAbs) have been developed as alternatives to conventional RIGs. Among them, the two-antibody cocktail comprising docaravimab and miromavimab is the only combination currently aligned with WHO guidelines for rabies PEP [9]. These antibodies target non-overlapping epitopes on the rabies virus glycoprotein and neutralize a broad range of wild-type isolates, including Indian field strains [10,11]. TwinRab™, a fixed-dose mAbs cocktail containing docaravimab and miromavimab, was developed by Cadila Healthcare Ltd. in collaboration with the WHO. It received Orphan Drug Designation from the US Food and Drug Administration (FDA) in May 2019 and was subsequently approved by the Drug Controller General of India (DCGI) for rabies PEP. Under the national Post-exposure Intervention for Neutralization and Dilution-Ready Use of Passive Immunization (PINDRUP) program, TwinRab™ aims to replace animal-derived RIGs with scalable, safe, and field-adaptable mAbs for public health use.

While mAbs address many of the supply and safety limitations associated with plasma-derived RIGs, their successful integration into routine practice also requires evidence of operational stability and flexibility. In many public-sector clinics in India, RIGs are commonly diluted in 0.9% sodium chloride to facilitate multi-site infiltration and to manage limited supply. While TwinRab™ is manufactured for scalable deployment, data on its in-use stability after dilution and storage at ambient temperature are limited. This is an important operational consideration, especially in field conditions where cold-chain continuity cannot always be ensured [12].

To address this knowledge gap, the present study was conducted in two complementary components. The first was an in vitro dilution stability assessment designed to evaluate the neutralizing potency of TwinRab™ following dilution in 0.9% sodium chloride and storage at room temperature (25 ± 2 °C) for 4-24 hours, simulating routine field-handling conditions. The second was an in vivo efficacy evaluation using a Syrian hamster rabies challenge model to determine the protective effectiveness of undiluted TwinRab™ administered six or 24 hours after viral exposure. Together, these investigations provide comprehensive evidence of TwinRab™’s stability and biological efficacy, supporting its potential for field-level implementation under the PINDRUP initiative and alignment with WHO-recommended rabies control strategies.

## Materials and methods

Study objective and experimental design

This study was conducted to evaluate two critical attributes of TwinRab™, a monoclonal antibody cocktail developed for rabies PEP: its in-use stability and neutralizing potency after dilution and its biological efficacy in a delayed post-exposure setting. The investigation comprised two distinct components. The first component was an in vitro dilution stability study conducted at the Zydus Research Centre (ZRC) to assess the neutralizing activity of TwinRab™ using a validated RFFIT against the laboratory-adapted CVS-11 rabies virus strain. This arm simulated operational handling conditions by evaluating samples diluted in 0.9% sodium chloride and stored at room temperature (25 ± 2 °C) for four and 24 hours.

The second component was an in vivo efficacy study employing a Syrian hamster rabies challenge model. Undiluted TwinRab™ was administered six hours or 24 hours post-virus challenge to assess its protective potential under delayed intervention timelines. This arm was designed to represent field-relevant post-exposure scenarios in rabies management.

Together, these complementary evaluations support the practical application of TwinRab™ under the national PINDRUP initiative and WHO-aligned passive immunization programs.

Product information and dilution protocols

TwinRab™ is a monoclonal antibody cocktail containing two human monoclonal antibodies - docaravimab and miromavimab - that bind to non-overlapping epitopes on the rabies virus glycoprotein, thereby neutralizing viral entry. The product is available in two strengths: 300 IU/mL and 600 IU/mL. Two GMP-manufactured batches were used in this study: Batch No. EXWHOBT0006H3P001 (600 IU/mL) and Batch No. EXWHOBT0006H4P001 (300 IU/mL).

For the in vitro stability evaluation, each batch was diluted in 0.9% sodium chloride to obtain twofold, fivefold, and 10-fold dilutions. Diluted samples were stored at ambient temperature (approximately 25 °C) and analyzed at four hours and 24 hours post preparation to assess short-term stability under simulated in-use conditions. Undiluted control samples were maintained at 2-8 °C to verify assay validity.

In vitro potency assessment

The neutralizing activity of diluted TwinRab™ was evaluated using the RFFIT in accordance with WHO-recommended protocols for potency testing of rabies immunoglobulins. The assay employed the CVS-11 laboratory-adapted rabies virus strain, with Baby Hamster Kidney (BHK-21) cells as the indicator cell line.

Each sample was incubated with a known quantity of virus (50 TCID₅₀) for neutralization, followed by inoculation onto pre-seeded BHK-21 monolayers. After a 20-22 hour incubation period, cells were fixed in acetone and stained using a fluorescein isothiocyanate (FITC)-labeled anti-rabies nucleoprotein antibody. The endpoint titer was defined as the dilution at which 50% of the microscopic fields showed complete inhibition of fluorescent foci. Potency was calculated in international units per millilitre (IU/mL) relative to the NIBSC reference standard. Results were expressed as mean ± standard deviation (SD) for each dilution and time point.

In vivo efficacy study

The in vivo efficacy of TwinRab™ was assessed in a Syrian golden hamster (*Mesocricetus auratus*) rabies challenge model. All experimental procedures were conducted in accordance with institutional ethical standards and approved by the Institutional Animal Ethics Committee (Protocol No. VAC/133/2011).

Animals were challenged intramuscularly in the left thigh with 0.1 mL of CVS-11 rabies virus suspension containing 50 LD₅₀, a dose known to produce 100% mortality in untreated controls. Following virus challenge, animals received either TwinRab™ or comparator human rabies immunoglobulin (Kamrab, HRIG) at six hours or 24 hours post exposure. Each animal received 15 IU of antibody administered intramuscularly at the site of virus inoculation.

The study included five groups (n = 10 animals per group): TwinRab™, six hours; Kamrab, six hours; TwinRab™, 24 hours; Kamrab, 24 hours; and a virus control group. Animals were monitored daily for 30 days for clinical signs of rabies. Humane endpoints were applied to animals showing progressive neurological signs or a moribund condition. Protection was defined as survival without the development of clinical rabies (Table 1).

**Table 1:** Study Groups for the In Vivo Efficacy Evaluation of TwinRab™

Group	Treatment	Dose (IU/animal)	Time of Administration Post-Challenge	n (%) (animals)
1	TwinRab™	15 IU	6 hours	10 (20%)
2	Kamrab (HRIG)	15 IU	6 hours	10 (20%)
3	TwinRab™	15 IU	24 hours	10 (20%)
4	Kamrab (HRIG)	15 IU	24 hours	10 (20%)
5	Virus Control (no treatment)	–	–	10 (20%)
Total	–	–	–	50 (100%)

Assay for potency and data analysis

The potency of TwinRab™ samples was quantified in IU/mL relative to the NIBSC reference standard, and data were expressed as mean ± SD. Statistical analysis was performed to determine potential differences in potency between four-hour and 24-hour samples. For the in vitro stability study, paired t-tests were used to compare mean potency values at the two time points for each dilution (twofold, fivefold, and 10-fold) and concentration (300 IU/mL and 600 IU/mL). For the in vivo efficacy study, survival proportions between treatment groups were compared using the chi-square (χ²) test.

A p > 0.05 was considered statistically significant. All analyses were performed using GraphPad Prism (version X; GraphPad Software, San Diego, CA) or equivalent statistical software.

## Results

In-use dilution stability of TwinRab™

TwinRab™ retained its specific biological activity following dilution in 0.9% sodium chloride and storage at room temperature for up to 24 hours. For both the 600 IU/mL and 300 IU/mL batches, measured potency values remained close to their theoretical values across all dilution levels (twofold, fivefold, and 10-fold) and time points (four hours and 24 hours).

Statistical analysis using paired t-tests revealed no significant loss of potency between four-hour and 24-hour samples for the 600 IU/mL batch, confirming that TwinRab™ remained stable under simulated in-use conditions. In contrast, for the 300 IU/mL batch, minor but statistically significant differences were observed at higher dilutions (fivefold and 10-fold), indicating a dose-dependent variation in stability. Control samples (undiluted and stored at 2-8 °C) consistently retained expected potency, confirming assay validity.

All potency results are expressed as mean ± SD in IU/mL for each dilution and time point (Tables 2-3).

**Table 2:** Potency of TwinRab™ 600 IU/mL Batch (EXWHOBT0006H3P001) After Dilution and Storage at Room Temperature

Dilution Condition	Theoretical Potency (IU/mL)	Control (IU/mL)	4 h Mean ± SD (IU/mL)	24 h Mean ± SD (IU/mL)	t-value	p-value
Control (undiluted)	600	646.26	–	–	–	–
Twofold dilution	300	–	369.34 ± 36.93	346.61 ± 34.67	−1.419	0.1731
Fivefold dilution	120	–	135.14 ± 12.67	134.70 ± 13.47	−0.075	0.9409
10-fold dilution	60	–	72.18 ± 6.90	71.94 ± 9.87	−0.063	0.9504

**Table 3:** Potency of TwinRab™ 600 IU/mL Formulation After Serial Dilution and ±25 % Variation in the Observed Range

Dilution Condition	Theoretical Potency (IU/mL)	Observed Range (±25%) (IU/mL)	Control (IU/mL)
Control (undiluted)	600	450-750	646.26
Twofold dilution	300	225-375	369.34
Fivefold dilution	120	90-150	135.14
10-fold dilution	60	45-75	72.18

**Figure 1:**
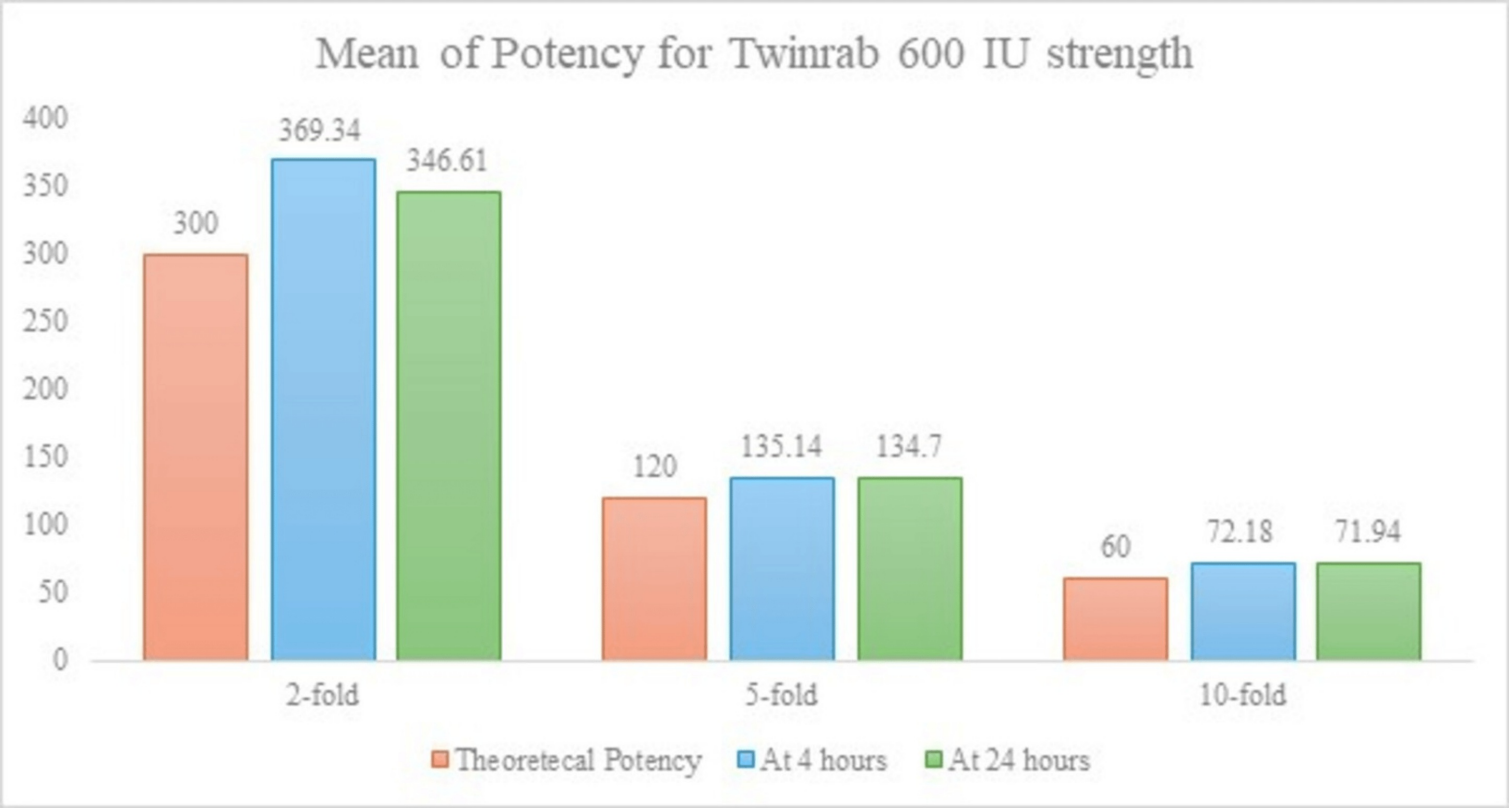
In-Use Stability of TwinRab™ 600 IU/mL After Dilution in 0.9% Sodium Chloride and Storage at Room Temperature No significant change in potency was observed across twofold, fivefold, and 10-fold dilutions over 24 hours (p > 0.05).

Control represents the mean observed potency under undiluted condition, while subsequent dilutions demonstrate theoretical and expected potency loss. All values are expressed in IU/mL. Figure 1 presents the in-use stability of TwinRab™ 600 IU/mL after dilution in 0.9% sodium chloride and storage at room temperature.

Across all dilutions, no statistically significant time-dependent change in potency was detected (p > 0.05), confirming excellent stability of the 600 IU/mL formulation over 24 hours at room temperature (Tables 4-5).

**Table 4:** Potency of TwinRab™ 300 IU/mL Batch (EXWHOBT0006H4P001) After Dilution and Storage at Room Temperature

Dilution Condition	Theoretical Potency (IU/mL)	Control (IU/mL)	4 h Mean ± SD (IU/mL)	24 h Mean ± SD (IU/mL)	t-value	p-value
Control (undiluted)	300	342.34	–	–	–	–
Twofold dilution	150	–	164.52 ± 15.30	168.93 ± 15.97	0.631	0.5363
Fivefold dilution	60	–	79.17 ± 7.91	70.13 ± 7.56	−2.713	0.0142
10-fold dilution	30	–	41.46 ± 4.67	46.53 ± 5.02	2.338	0.0311

**Table 5:** Potency of TwinRab™ 300 IU/mL Formulation Following Serial Dilution up to 10-Fold and ±25% Acceptable Variation

Dilution Condition	Theoretical Potency (IU/mL)	Observed Range (±25%) (IU/mL)	Control (IU/mL)
Control (undiluted)	300	225-375	339.34
Twofold dilution	150	112.5-187.5	169.67
Fivefold dilution	60	45-75	67.87
10-fold dilution	30	22.5-37.5	33.93

**Figure 2:**
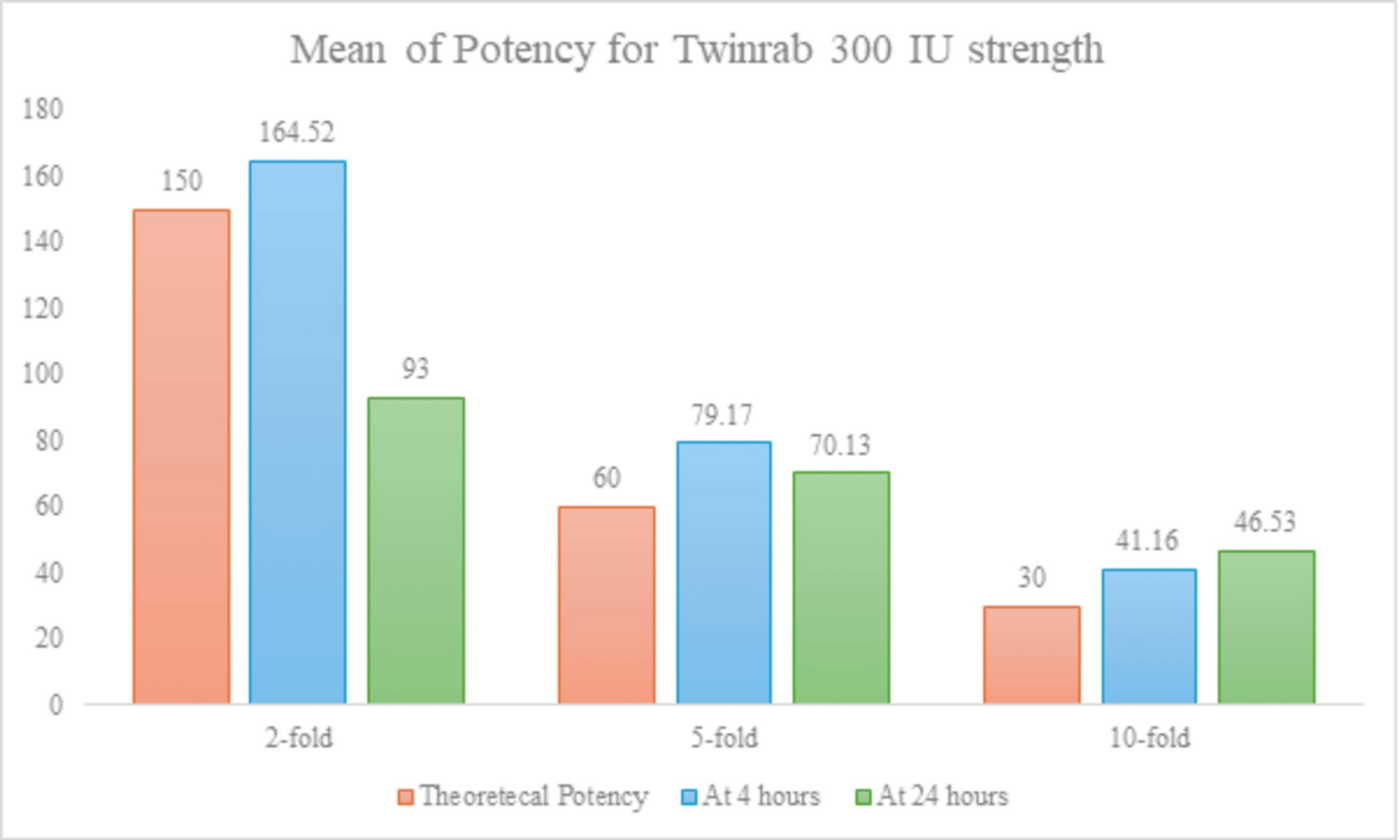
In-Use Stability of TwinRab™ 300 IU/mL After Dilution in 0.9% Sodium Chloride and Storage at Room Temperature Minor but significant potency changes occurred at higher dilutions (p < 0.05), remaining within acceptable limits.

Control denotes undiluted sample potency. Calculated “Control IU/mL” values are derived proportionally from the 600 IU reference batch. All potencies are expressed as IU/mL. Figure 2 presents the in-use stability of TwinRab™ 300 IU/mL after dilution in 0.9% sodium chloride and storage at room temperature.

For the 300 IU/mL batch, slight differences were noted at fivefold and 10-fold dilutions, indicating minor dose-dependent variation in potency over 24 hours. Nevertheless, the observed differences were within acceptable biological limits, confirming that TwinRab™ maintained its functional stability under practical handling conditions.

In vivo protection following post-dilution administration of TwinRab™

The in vivo efficacy of TwinRab™ was evaluated in a Syrian hamster model of rabies virus challenge. At six hours post challenge, TwinRab™ 15 IU conferred 90% survival (9/10 animals) compared with 50% survival (5/10) for Kamrab 15 IU. Although this 40% difference approached statistical significance (p = 0.0571), it suggests a faster onset of protective action for TwinRab™.

**Figure 3:**
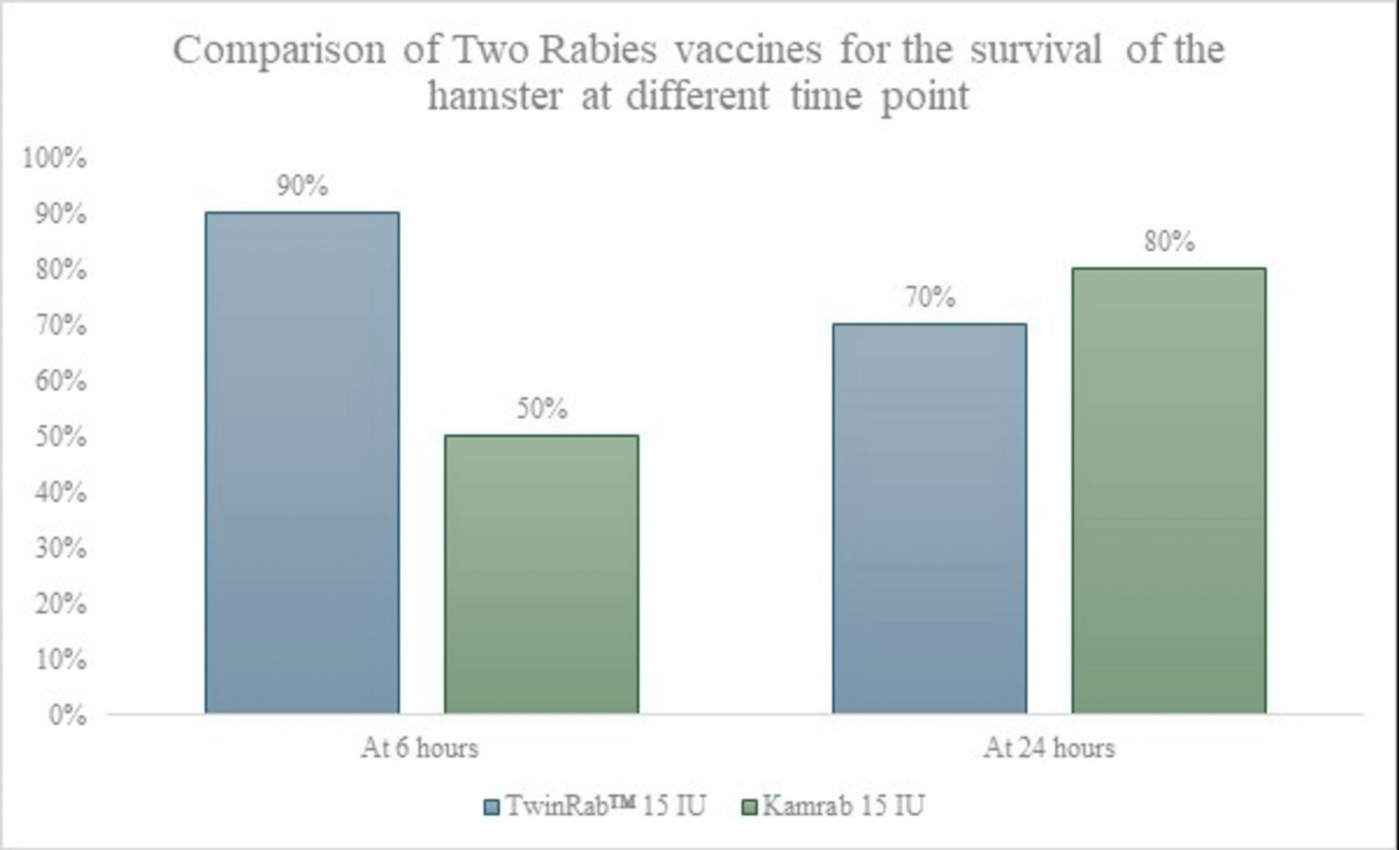
Comparative Post-exposure Protection in Hamsters Treated With TwinRab™ or Kamrab

At 24 hours post challenge, both preparations demonstrated comparable protection (TwinRab™, 70%; Kamrab, 80%; p = 0.6147). All animals in the untreated virus-control group succumbed to infection, confirming the lethality of the challenge dose and the validity of the model (Table 6). Figure 3 presents the comparative post-exposure protection in hamsters treated with TwinRab™ or Kamrab.

**Table 6:** Survival of Hamsters Treated With TwinRab™ and Kamrab Following CVS-11 Challenge

Treatment Group	Time Point	Survival n/N (%)	χ² Value	p-value
TwinRab™ 15 IU	6 h	9/10 (90%)	3.619	0.0571
Kamrab 15 IU	6 h	5/10 (50%)	–	–
TwinRab™ 15 IU	24 h	7/10 (70%)	0.253	0.6147
Kamrab 15 IU	24 h	8/10 (80%)	–	–
Virus Control	–	0/10 (0%)	–	–

By 24 hours, both treatments achieved similar overall protection, indicating equivalent efficacy at later stages but differing onset kinetics.

## Discussion

The present study evaluated the in-use stability and biological efficacy of TwinRab™, a two-antibody monoclonal cocktail (docaravimab + miromavimab) developed for rabies PEP. The in vitro data demonstrated that TwinRab™ retained its specific biological activity following dilution in 0.9% sodium chloride and storage at room temperature for up to 24 hours. For the 600 IU/mL batch, mean potency values across all dilution levels (twofold, fivefold, and 10-fold) showed no statistically significant difference and remained consistent between the four-hour and 24-hour time points, indicating excellent temporal stability under simulated in-use conditions. While the 300 IU/mL batch exhibited slight variations at higher dilutions, these changes were minor and remained within the acceptable biological limits. This indicates that the potency was preserved across all tested conditions. Together, these findings confirm that TwinRab™ maintains functional stability across operationally relevant concentrations and time frames, supporting its use in real-world clinical settings where immediate cold-chain access may be limited.

These observations are particularly relevant in India, where rabies remains endemic and continues to cause an estimated 18,000-20,000 deaths annually [13,14]. Despite national guidelines recommending the combined use of vaccine and RIG for category III exposures, HRIG and ERIG remain under-utilized because of donor dependence, variable potency, cold-chain requirements, and cost constraints. The ability of TwinRab™ to retain potency after dilution and storage at ambient temperature directly addresses these programmatic barriers. Its standardized composition and batch-to-batch consistency provide practical advantages over plasma-derived products and align with the WHO goals of ensuring equitable access to effective passive immunization.

The biological activity of TwinRab™ is conferred by its constituent monoclonal antibodies, docaravimab and miromavimab, which bind to distinct, non-overlapping epitopes on the rabies virus glycoprotein [15,16]. This dual-target mechanism restricts viral entry and reduces the likelihood of escape through antigenic mutation, offering a neutralization spectrum comparable to, or broader than, conventional polyclonal immunoglobulins. The preserved neutralizing potency observed at multiple dilutions and time points is consistent with the high physicochemical stability reported for other monoclonal antibody-based therapeutics [17]. From an operational standpoint, this characteristic is particularly valuable in resource-limited healthcare settings, where dilution and short-term storage are common practices used to enable multi-site wound infiltration and optimize product utilization.

The in vivo arm of this study further substantiates the functional relevance of TwinRab™ stability. In the Syrian hamster challenge model, TwinRab™ administered six hours post exposure achieved a 90% survival rate, compared with 50% for HRIG (Kamrab). This outcome suggests that the early protective response with TwinRab is robust, which may be particularly advantageous in post-exposure scenarios where rapid viral neutralization is critical. At 24 hours post challenge, both TwinRab™ and Kamrab demonstrated comparable protection (70% vs 80%, respectively), indicating equivalent overall efficacy at later intervention points. These results reinforce that TwinRab™ can provide a better and effective early and sustained protection consistent with the WHO-recommended PEP framework.

The current findings collectively highlight TwinRab™ as a stable, efficacious, and field-adaptable monoclonal antibody preparation suitable for integration into public health programs such as the PINDRUP initiative. Its retained potency following dilution, stability at ambient temperature, and robust protective efficacy support decentralized use in peripheral health facilities without strict dependence on cold-chain infrastructure. This operational flexibility can help expand passive immunization coverage in high-burden, resource-constrained regions and contribute to the broader goal of eliminating dog-mediated human rabies deaths by 2030.

The study demonstrates robust evidence for the in-use stability and biological efficacy of TwinRab™, yet some methodological constraints merit acknowledgment. The in vitro stability assessment utilized the laboratory-adapted CVS-11 rabies virus strain, which is the standard and regulatory-approved strain for such assays. However, as CVS-11 represents a reference laboratory strain, it may not fully capture the antigenic variability observed among field isolates circulating in India. In addition, the Syrian hamster challenge model, although widely accepted for rabies research, reflects a controlled preclinical setting and may not encompass the full complexity of human exposures. Future studies incorporating region-specific viral strains and clinical evaluations under field conditions would strengthen the translational relevance of these findings. Nevertheless, the demonstrated stability and retained neutralizing potency of TwinRab™ across multiple dilutions and time points reaffirm its potential as a next-generation passive immunization product that combines scalability, thermostability, and operational practicality, effectively addressing key limitations of current rabies PEP delivery.

Twinrab demonstrates non-inferior potency compared to theoretical standards across all tested dilutions and time points. At a twofold dilution (theoretical potency: 300 IU/mL), TwinRab™ shows 369 IU/mL at four hours and 346 IU/mL at 24 hours, both falling within acceptable variability limits (p > 0.05). At a fivefold dilution (theoretical potency: 120 IU/mL), TwinRab™ records 135 IU/mL at four hours and 134 IU/mL at 24 hours, confirming non-inferiority (p > 0.05). Similarly, at a 10-fold dilution (theoretical potency: 60 IU/mL), TwinRab™ achieves 72 IU/mL at four hours and 71 IU/mL at 24 hours, further demonstrating consistent non-inferior potency across conditions (p > 0.05).

These results support the conclusion that TwinRab™ maintains its biological potency across dilutions and time points. This is consistent with the expected performance of cell-based viral potency assays, which are known to carry an acceptable day-to-day variability of approximately ±20% due to inherent biological and procedural factors.

As per the results in our study, in a special clinical case scenario, alone wherein there are multiple scattered category 3 bite wounds and the volume of infiltration of Twinrab is calculated as per 40 IU/kg approved dosage, Twinrab should be diluted as follows - Given 1 ml of Twinrab needs to be administered, it can be diluted 10 fold with normal saline for usage in rabies post exposure prophylaxis of multiple scattered wounds. The clinician may use a 10 ml syringe and aspirate 9 ml of NS and then aspirate 1 ml of Twinrab from the vial to administer the same in a category 3 bite wound as per intra-lesion transfer. This will lead to an effective potency of 72IU/ml even after 10 fold dilution, as per this study.

## Conclusions

TwinRab™ maintained its functional potency and biological efficacy under conditions simulating routine clinical use. The in vitro stability assessment confirmed consistent neutralizing activity following dilution in 0.9% sodium chloride and storage at room temperature for up to 24 hours, with no significant loss of potency across most dilutions. The in vivo efficacy evaluation further demonstrated that undiluted TwinRab™ provided substantial protection when administered six or 24 hours after rabies virus challenge, showing outcomes comparable to conventional HRIG. Collectively, these results establish TwinRab™ as a stable, scalable, and field-adaptable monoclonal antibody preparation suitable for inclusion in national rabies control programs. Its thermostability and operational flexibility make it particularly relevant for deployment in resource-limited, high-incidence regions where cold-chain interruptions and treatment delays continue to hinder effective PEP.
